# Application-Oriented Data Migration to Accelerate In-Memory Database on Hybrid Memory

**DOI:** 10.3390/mi13010052

**Published:** 2021-12-29

**Authors:** Wenze Zhao, Yajuan Du, Mingzhe Zhang, Mingyang Liu, Kailun Jin, Rachata Ausavarungnirun

**Affiliations:** 1 School of Computer and Artificial Intelligence, Wuhan University of Technology, Wuhan 430070, China; wenze_smile@163.com (W.Z.); lmy569@whut.edu.cn (M.L.); wnnnntnl@whut.edu.cn (K.J.); 2 Shenzhen Research Institute of Wuhan University of Technology, Shenzhen 518000, China; 3 Institute of Computing Technology, Chinese Academy of Sciences, Beijing 100190, China; zhangmingzhe@iie.ac.cn; 4 Sirindhorn International Thai-German Graduate School of Engineering, King Mongkut’s University of Technology North Bangkok (KMUTNB), Bangkok 10800, Thailand; r.ausavarungnirun@gmail.com

**Keywords:** in-memory database, hybrid memory, data migration

## Abstract

With the advantage of faster data access than traditional disks, in-memory database systems, such as Redis and Memcached, have been widely applied in data centers and embedded systems. The performance of in-memory database greatly depends on the access speed of memory. With the requirement of high bandwidth and low energy, die-stacked memory (e.g., High Bandwidth Memory (HBM)) has been developed to extend the channel number and width. However, the capacity of die-stacked memory is limited due to the interposer challenge. Thus, hybrid memory system with traditional Dynamic Random Access Memory (DRAM) and die-stacked memory emerges. Existing works have proposed to place and manage data on hybrid memory architecture in the view of hardware. This paper considers to manage in-memory database data in hybrid memory in the view of application. We first perform a preliminary study on the hotness distribution of client requests on Redis. From the results, we observe that most requests happen on a small portion of data objects in in-memory database. Then, we propose the Application-oriented Data Migration called ADM to accelerate in-memory database on hybrid memory. We design a hotness management method and two migration policies to migrate data into or out of HBM. We take Redis under comprehensive benchmarks as a case study for the proposed method. Through the experimental results, it is verified that our proposed method can effectively gain performance improvement and reduce energy consumption compared with existing Redis database.

## 1. Introduction

With the vigorous development of cloud computing and Internet technologies, a large number of emerging applications with high concurrency and low latency have emerged. They put forward new challenges to the performance of traditional computer systems. Due to the access speed limitation, it is difficult for disk storage-based database system to meet the scalability and delay requirements of such new applications. As a result, in-memory database system, such as Redis [1] and Memcached [2], becomes an excellent solution and has been widely applied in today’s data centers, such as E-commerce websites, game applications and social networking sites.

In-memory database stores data in memory instead of disk drives to achieve faster response times by eliminating the need to access disks. Its performance can be significantly affected by the speed of reading and writing data in memory. However, conventional DRAM faces the bottleneck of limited bandwidth due to the scalability of pin count [3], which is commonly known as the memory wall [4]. The advent of die-stacked memory (e.g., HBM [5], Hybrid Memory Cube (HMC) [6]) has become a promising solution [7] to alleviate the memory wall problem by delivering several orders of magnitude higher bandwidth and lower energy consumption. Unfortunately, the capacity of die-stacked memory is significantly smaller because of limited number of stacks and the interposer challenges [8]. Thus, die-stacked memories are often used as a part of main memory or cache to form a hybrid memory together with traditional DRAM memory.

There have been existing studies on die-stacked memory-based hybrid memory. How to place and migrate data between two types of memories is mostly studied to optimize performance and energy efficiency. Vasilakis et al. [9] propose to exploit last level cache (LLC) to detect memory segment with high data locality for migration. Sim et al. [10] propose to leverage a customized hardware-managed remapping table to keep page remapping without operating system interventions. Prodromou et al. [11] introduce a dynamic memory manager that groups existing memory controllers into several Pods and inserts them between LLC and the system’s memory controllers. These works all consider the data migration in the view of hardware management.

In this paper, we consider in-memory database data management on hybrid memory system in the view of application. To best exploit respective advantages and overcome drawbacks for performance gain in hybrid memory system, adding migration support between the two types of memory is of great importance. There are several challenges facing the design of such migration scheme. First, providing efficient application-level interfaces related to memory allocation and free is critical for establishing connection between upper application and lower device. By utilizing these interfaces, users can easily control these two memories from the perspective of application. Furthermore, it is a key point to determine the appropriate migration granularity. Different from the page or block granularity at device-level, the granularity at application-level needs to be considered with combinations of specific semantics of the application. Finally, identifying what to be placed in which type of memory is an important task that is directly related to the efficient use of space and performance gains.

As a result, in this paper we focus on exploring an efficient application-level migration policy for in-memory database systems that executes hybrid memory systems with flat memory organization. We first perform a preliminary study on Redis in-memory database and use its benchmarking tool to simulate client requests on data objects. From the study results, we observe that a large portion of requests happen on a small amount of data objects, which indicates the characteristics of hot data. By exploiting this observation, we propose an Application-oriented Data Migration method called ADM to accelerate the in-memory database based on hybrid memory architecture. The key idea is to migrate hot data objects in the database to HBM die-stacked memory for high bandwidth. We design a hotness management scheme and two migration policies to migrate into and out of HBM, respectively. By extending the existing *malloc* and *free* interface for main memory to be *HBM_malloc* and *HBM_free*, we integrate the ADM migration method into a traditional in-memory database for programmers.

In order to evaluate the efficiency of the proposed ADM, we conduct comprehensive experiments on Redis, a commonly used in-memory database, under various client benchmarks generated by the memtier_benchmark tool. Experimental results indicate that our proposed migration method can significantly optimize system performance as well as energy consumption. In summary, our contributions in this paper are listed as follows:

- Observation of the hotness characteristics of data objects in in-memory database from a preliminary study.
- By exploiting the observation, we propose the application-oriented migration method ADM, which contains a data hotness management scheme and two migration policies.
- We implement the proposed application-oriented data migration into Redis, and evaluate its performance under comprehensive benchmarks. Experimental results have verified its effectiveness on system performance and energy consumption.

The remainder of the paper is organized as follows. We present the background and motivation for this work in Section 2. Section 3 describes the details of our proposed ADM. Evaluation results are shown and discussed in Section 4. We conclude the paper and future planned work in Section 5.

## 2. Background and Motivation

In this section, we first present the basics of in-memory database and die stacked memory-based hybrid memory. Then, we investigate the characteristics of the in-memory database system in terms of object hotness. At last, motivation of this paper is presented.

### 2.1. In-Memory Database

**Figure 1:**
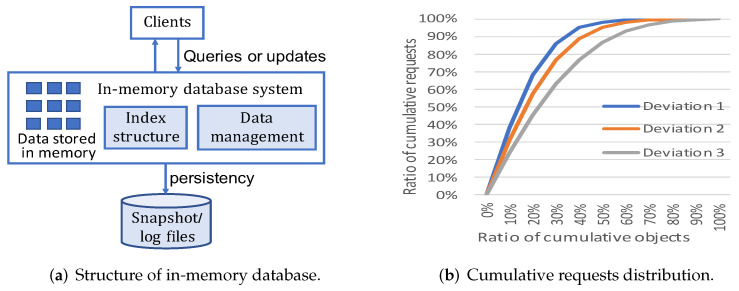
The background of in-memory database and request distributions of objects under Gaussian distributions with three deviations.

In-memory database is a database system that mainly relies on main memory to store data [12]. Figure 1a shows the basic architecture of the in-memory database. Clients send query and update requests to access data in the database. Data are stored in memory in a directly usable format without the barrier of compression or encryption [13]. In-memory database systems often exploit index structure to accelerate response speed of data queries. Due to the volatility of memory, in-memory database systems require persistency mechanisms, e.g., creating logs or snapshot files in disks, to avoid data loss upon power outage or server failure.

Mainstream in-memory databases are mainly categorized into key-value in-memory database, relational in-memory database, and other databases. Among them, the key-value in-memory database performs data access operations by keys. The values usually support various data types. The key-value data structure is simple and flexible, which is especially suitable for applications that have different kinds of data structures. Typical key-value in-memory databases include Redis [1], Memcached [2] and Aerospike [14]. As a representative in-memory database, Redis is an open source, networked, and single-threaded database system. Key-value data in Redis use a dictionary to establish the mapping from key to value. In particular, the values support several types of data objects, including *String*, *List*, *Zset*, *Hash*, etc. This paper considers Redis as the case study of our proposed method for in-memory database.

### 2.2. Hybrid Memory System

Although die-stacked memory has the similar memory cell arrays and peripheral logic to conventional DRAM, it can provide higher access bandwidth and lower energy by using die-stacked techniques. This paper mainly considers HBM die-stacked memory. Its special through silicon vias (TSVs) structure enables wider I/O interfaces (I/F) among vertically stacked layers. By integrating hundreds of TSVs through layers, HBM can provide a much higher memory channel bandwidth (BW) [15]. However, due to the interposer challenge, its capacity expansion is limited. Table 1 shows the performance and energy comparisons among two DRAM types (DDR4 and DDR5) and two HBM types (HBM3 [16] and HBM2) [17]. Although HBM can provide a much higher bandwidth and lower energy, its capacity is limited and cannot be used as main memory independently. Thus, the hybrid memory is designed to exploit advantages of DRAM and HBM in bandwidth and capacity.

In hybrid memory, HBM has been considered as two hybrid modes of cache, part of main memory or both. This paper considers to combine HBM and DRAM together to form the main memory in flat address-space. Existing works have studied the data migrations between two types of memories to achieve high performance. LGM [9] leverages LLC to identify memory segments with high spatial and temporal locality for the selection of migrated data. SILC-FM [18] designs a hardware data management mechanism to organize stacked memory as an associative structure and allow interleaved subblock placement. The re-configurable hybrid memory has been proposed, such as the Intel Xeon Phi KNL architecture [19] that supports two hybrid-modes configurations. CHAMELEON [20] proposes a hardware-software co-design to dynamically switch memory regions between two hybrid modes according to the status of free space. Hybrid2 [21] exploits a small portion of HBM stacked memory as cache, and minimizes the metadata overheads by extending an on-chip tag. These works consider hybrid memory management in view of the hardware.

In this paper, we consider managing the data of the in-memory database in hybrid memory from the perspective of applications. For device-level data management, inherent memory regions, such as page and block, are usually directly used as design objects. Furthermore, it relies more on the significant modifications of the hardware architecture. For example, page table entry(PTE), translation lookaside buffer(TLB) and caches are usually utilized to achieve design goals. Differently, for our application-level data management, the information about the underlying device is not visible and can not be exploited by users. We need to extract useful information on target application based on the study of its characteristics. Then, the modification of application’s source code is necessary for the implementation of scheme. Moreover, the application layer is closer to user compared with lower device layer. In short, the specific design measures for application- and device-level, including design object, design granularity, implementation principle, etc., are entirely different.

### 2.3. Motivation and Scope of This Paper

In this section, we perform a preliminary study on data objects characteristics of the Redis database. We use the memtier_benchmark, a high-throughput benchmarking tool for Redis and Memcached to generate client requests [22]. 10 million requests on 100 thousand objects are generated. We collect the amount of requests that access on objects. Then, the object hotness distribution is collected and shown in Figure 1b. We specify the Gaussian distribution mode in memtier_benchmark and adopt three standard deviation configurations including Deviation 1, Deviation 2 and Deviation 3 with 10%, 12.5% and 16.6% of object numbers. From the curves in Figure 1b, we can observe that a great amount of workload’s requests are distributed on a small number of data objects in in-memory database. For instance, almost 30% objects occupy 90% requests accesses in Deviation 1.

**Our consideration.** Motivated by above hotness observation results, we consider to manage data objects in the in-memory database on the hybrid memory system. As HBM has a much higher bandwidth, those hot (frequently-accessed) objects that require fast access speed would be more suitable to be stored in HBM. Keeping this in mind, we propose to build an application-level hotness-aware data migration scheme.

**Scope of this paper.** Our whole work is conducted from the perspective of the application layer, directly modifying the original in-memory database application software code. On the basis of the original code, a new migration mechanism is added for data management of the in-memory database based on the new hybrid memory architecture. Hardware details such as parallelism and pipelines are not involved. In addition, our purpose is to optimize performance and energy consumption of in-memory database applications on hybrid memory system. Its fault tolerance and reliability are out of our research scope.

## 3. Design and Implementation

In this section, we first present a high-level overview of our proposed ADM method, and then introduce the detailed designs of its three components. At last, the implementation of ADM in Redis in-memory database is illustrated.

### 3.1. Overview

**Figure 2:**
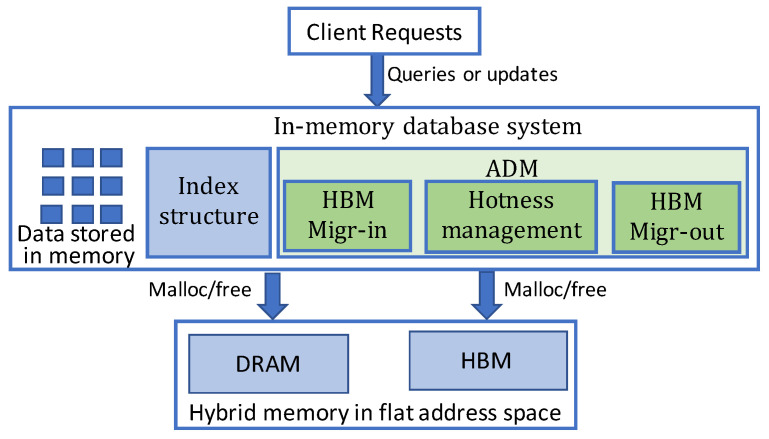
Overview of the proposed ADM method. ADM contains three components and is integrated to the Redis in-memory database.

Our proposed ADM approach aims at high-performance in-memory database on hybrid memory in the application level. ADM mainly consists of three components: Hotness management, HBM Migr-in and HBM Migr-out, as shown in Figure 2. The HBM Migr-in component is responsible to migrate hot data objects of in-memory database from DRAM to HBM, while the HBM Migr-out component is in charge of the migration of cold data from HBM to DRAM. The hotness management component monitors and manages the hotness of data objects both in DRAM and HBM. For DRAM, a least frequently used (LFU) hotness management policy is applied on data objects. In order to decouple the hotness property when objects are migrated into HBM, we design a novel counter based hotness management scheme for HBM data objects. Hotness management works together with the other two components to provide the hotness information and adjust object hotness according to the management policy.

### 3.2. Hotness Management

Since the limited capacity in HBM and migration overhead, the decision about whether an object should be migrated to HBM must be carefully considered. For objects in DRAM, Redis records data object hotness by maintaining a value field for each object. It uses the LFU algorithm to indicate how often the object is accessed and evict the victim object for persistent storage. A lfu-log-factor field is used to adjust the increase speed of hotness, while a lfu-decay-time field is used to adjust the hotness decrease speed. In our hotness management for DRAM, we use the existing LFU algorithm to find the hot data. By setting a hotness threshold, the migration out of DRAM would be invoked.

Due to the limited capacity of HBM, it is necessary to reclaim memory space of cold data objects in HBM for new coming objects migrated out of DRAM. For objects in HBM, hotness management establishes a counter for each object. When an object is accessed by a client request, its counter would increase by one. To prevent the counter from increasing too much, hotness management designs a hotness decay algorithm to limit the growth of counters. The algorithm would decay the counter value of each object with a fixed time interval. Specifically, when the decay is invoked periodically, counters of data objects would be halved. When the hotness counter threshold is satisfied, the object would be migrated out of HBM to make room for new objects.

### 3.3. HBM Migr-In

**Figure 3:**
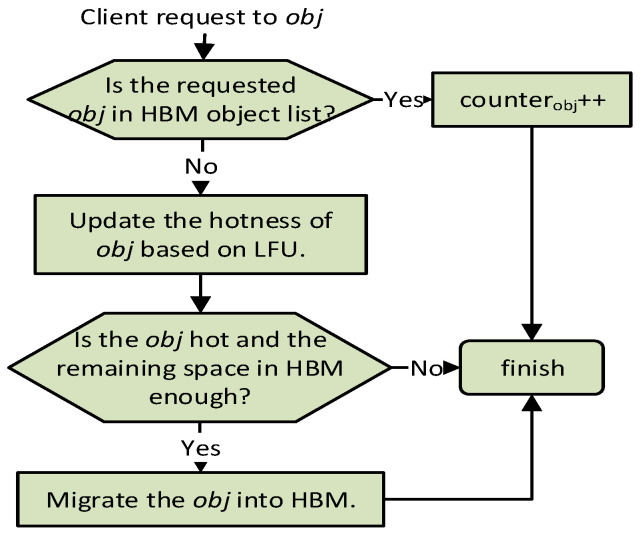
The migration workflow of HBM Migr-in from DRAM to HBM.

Figure 3 describes the workflow of object migration from DRAM to HBM managed by the HBM Migr-in component. It develops a threshold-based mechanism to identify whether an object is hot or not. The HBM Migr-in triggers the workflow when clients send a request to in-memory database. ADM maintains an object list to store all the objects in HBM. HBM Migr-in first checks whether the requested object is already in the object list of HBM. For objects in HBM, it would only increase their counter values by one. Otherwise, the hotness of the object is first updated according to the LFU rule. Then, the hotness of the object would be checked through comparing with the pre-set threshold $T_{in}$. If it exceeds the threshold and there is enough space in HBM, the object would be migrated into HBM. When HBM does not have enough space, the migration operation would be aborted. The migration from DRAM into HBM mainly includes the following five steps:

1. malloc space in HBM for the object the size of which is obtained from its metadata;
2. copy the value data and metadata of the object into HBM;
3. free the space of the migrated object in DRAM;
4. set an initial counter value for the object according to the counter-based hotness management;
5. insert an new entry to the object list in HBM.

### 3.4. HBM Migr-Out

HBM Migr-out reuses the lru field to store a counter value that counts accesses of objects stored in HBM. The counter increases for each request to the object. Our HBM Migr-out adopts an active, interval-based approach to manage object migration from HBM to DRAM.

**Figure 4:**
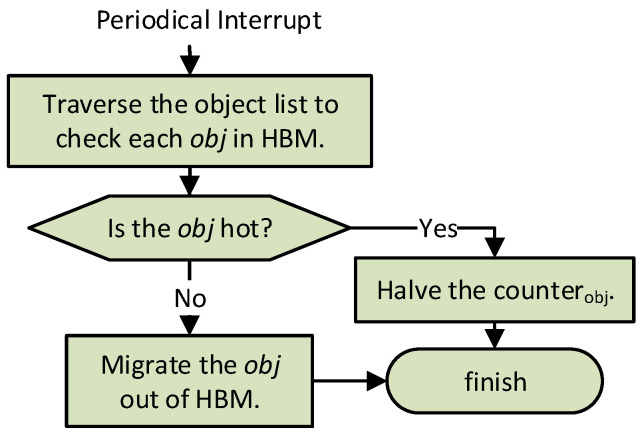
The migration workflow of HBM Migr-out from HBM to DRAM.

As described in Figure 4, HBM Migr-out periodically traverses the object list in HBM to check each object. For each object in HBM, Migr-out compares its counter value with the pre-set threshold $T_{out}$. If it is lower than the threshold, the object will be migrated out of HBM; otherwise the counter value will be halved for hotness updating. The migration out of HBM mainly includes the following five steps:

1. malloc space in DRAM for the object the size of which is obtained from its metadata;
2. copy the value data and metadata of the object into DRAM;
3. free the space of the migrated object in HBM;
4. set an initial LFU value for the object according to the LFU-based hotness management;
5. delete the corresponding entry from the object list in HBM.

### 3.5. Implementation of ADM

In order to implement the proposed ADM in existing DRAM-based memory system, we divide the main memory into two parts to simulate the HBM and DRAM space. The HBM is mapped into a contiguous range of address space, while the DRAM is mapped to another contiguous range in the same address space. Data objects are exclusively stored in HBM or DRAM address space, i.e., two memories are in flat address space and there is only one copy for each data object of in-memory database. In hybrid memory system, we assume that the DRAM resource is always sufficient for workloads while HBM has limited capacity. The page replacement with external storage is not under our considerations.

ADM can be integrated into the source code of in-memory database. We develop new Application Programming Interfaces (APIs) for memory allocation. For example, *hbm_malloc* and *hbm_free* are used to allocate and free HBM memory space, respectively. Therefore, programmers can directly exploit them to perform data management in different memories in the application level. In this way, data objects can be migrated between DRAM and HBM at runtime, without any changes of hardware management.

As a case study, this paper has already implemented ADM in Redis. As Redis stores data in the form of key-value pairs, and clients often query or update different types of values, such as *String*, *List* and so on. Our migration mechanisms have been applied onto these types of data objects.

## 4. Experiment and Evaluation

In this section, we present the evaluation of our proposed ADM method. We first introduce experiment settings, and then quantify the behaviors of migrations on the application data objects. At last, experimental results are illustrated in terms of system performance and energy consumption.

### 4.1. Experimental Setup

In order to evaluate the effectiveness of the ADM method on in-memory database, our experiment is performed in software and hardware parts. In the software part, the Redis in-memory database is running on a real computer configured with a 4-core CPU processor with the frequency of 2.5 GHz. We use a fast and scalable multi-core simulator, Zsim [23], to capture the memory trace of Redis. In the hardware part, we use a memory simulator, DRAMsim3 [24], to model the timing parameters and memory controller behaviors for the hybrid memory with HBM and DRAM. The support for flat address space hybrid memory composed of DRAM and HBM die-stacked memory is also implemented by extending DRAMsim3. Its detailed configurations are listed in Table 2.

**Figure 5:**
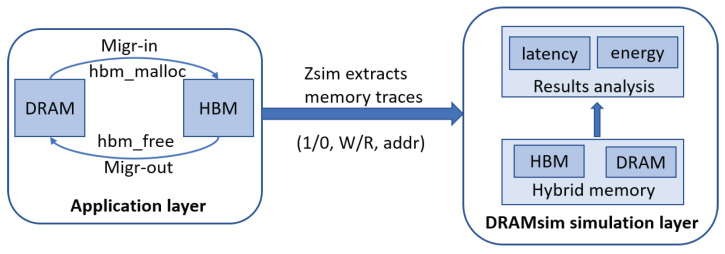
The modeling architecture and process of our experiment.

The modeling involved in our experiment is shown in Figure 5. As our scheme is application-oriented, the ADM is mainly implemented at the application layer. The hybrid memory simulation is mainly achieved by modifying the DRAMsim simulator [24]. As shown in the Figure 5, by extending new application program interfaces, we implement two kinds of memory spaces at the application layer and perform data migration operations between them. We use the Zsim simulator [23] to extract the memory read/write behaviors during the running process to form a trace file. Among them, each trace contains three parts: (1) Tag, in which “0” represents a request on DRAM, while “1” represents a request on HBM; (2) Request type, in which“W” stands for write request, while “R” stands for read request; (3) Access address, in which the access address of each request is stored. In our simulation process, Zsim first translates the virtual address obtained from the application layer into a physical address and stores it in the trace. Then, the trace file will be injected into the DRAMSim simulator to simulate the hardware behaviors in hybrid memory. Finally, we obtain the latency and energy consumption results of the hybrid memory from the simulation on DRAMsim.

In our experiments, Redis in version 5.0.8 in standalone mode is taken as a case study for in-memory database. All workloads are generated by memtier_benchmark, a command line utility developed by Redis Labs for load generation and benchmarking NoSQL key-value databases [22]. We construct five kinds of workloads with different ranges of data size and operation ratios (set-get) as listed in Table 3.

To quantify the impact on performance and energy consumption, we run Redis in-memory database under the following four memory types with different methods:

- **DRAM**: Redis runs on the single memory only including DRAM memory.
- **HM-W/O-Migr**: Redis runs on the hybrid memory architecture, but there is no migration scheme. Data objects enter in HBM in a first-come first-served way.
- **HM-W-Migr**: Redis integrated with our proposed ADM method runs on the hybrid memory architecture and there are migrations between DRAM and HBM.
- **HBM**: Redis runs on the single memory only including HBM memory type. We assume an ideal situation that the capacity of HBM memory is unlimited.

### 4.2. Behaviours of Object Migration

**Figure 6:**
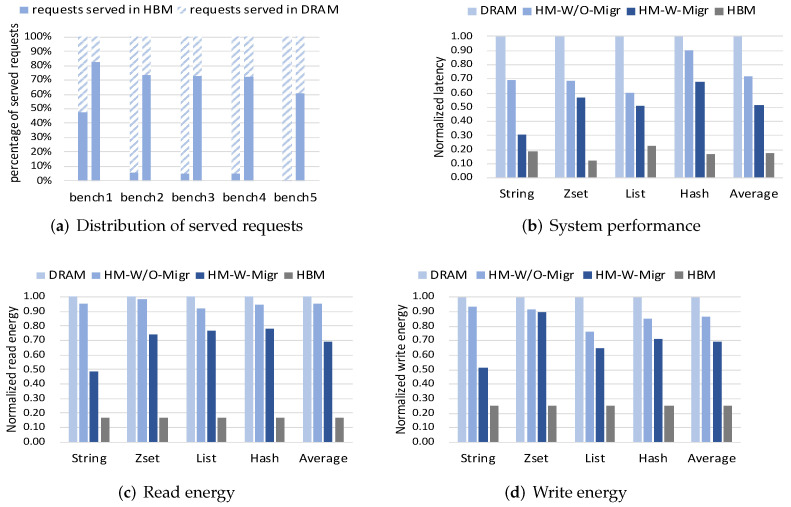
Distribution of served requests on application-level (**a**) and results of four methods on different data structures of Redis (**b**–**d**).

We first analyze the served behaviours of client requests in HBM/DRAM from the application perspective. Figure 6a shows the distribution of client requests served in HBM or DRAM across five workloads. The left column denotes the HM-W/O-Migr while the right column denotes the HM-W-Migr. From the figure, it can be found that the proposed ADM method can increase the percentage of client requests served in HBM. It implies the placement of hot data in faster HBM for serving more client requests. This behaviour shows that our proposed ADM method truly identifies the hot data and utilizes HBM space more frequently.

### 4.3. Performance and Energy Results

In this section, we first present the performance and energy results of data structures including *String*, *Zset*, *List* and *Hash*, as shown in Figure 6b–d. All results are obtained from Dramsim3 and normalized to Redis on DRAM. Performance refers to the latency to serve memory requests. As existing benchmarks do not support data structures besides to *String* [25], we extend the memtier_benchmark tool to support them.

From the performance results, We can see that HM-W-Migr outperforms the DRAM and HM-W/O-Migr in all workloads. On average, HM-W-Migr improves performance by 48% compared with the DRAM, and by 20% compared with the HM-W/O-Migr. From the energy results, we can find that HM-W-Migr can reduce read and write energy by 26% and 18% over HM-W/O-Migr on average, respectively. The HBM version in all figures shows ideal best results, while HM-W-Migr performs closest to the ideal case. Besides, we can observe that the ADM on *String* data can achieve the most improvement when compared with other data structures.

### 4.4. Sensitivity Analysis

In order to further verify the sensitivity of ADM, we investigate its effectiveness on different data size ranges and operation ratios between sets and gets on the *String* data type.

#### 4.4.1. Sensitivity to Data Size Range

**Figure 7:**
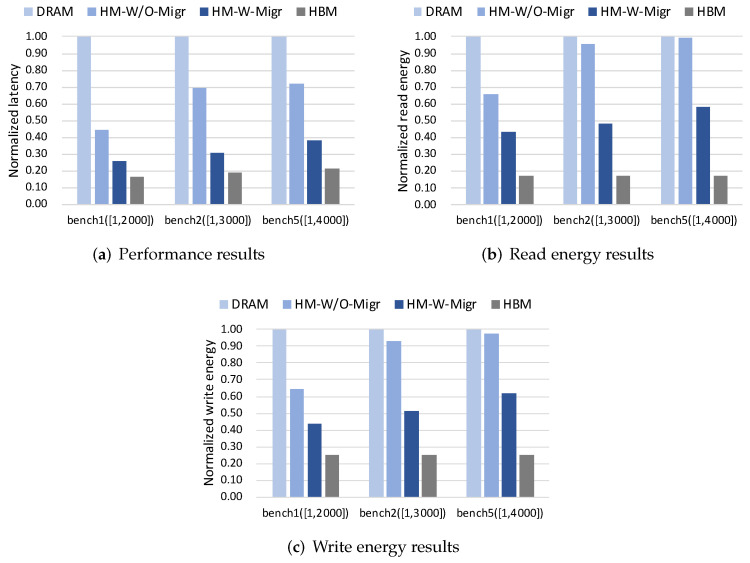
Normalized results across different size ranges.

**Figure 8:**
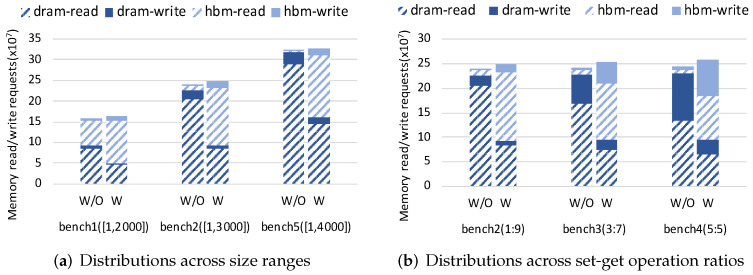
Distributions of dram/hbm read/write requests across different size ranges and set-get operation ratios.

Figure 7a–c show results of performance, read energy and write energy across three different data size ranges, respectively. From the results, we can find that when the data size increases, the performance and energy improvement of HM-W/O-Migr and HM-W-Migr both decrease compared with the DRAM case. However, compared to HM-W/O-Migr that drops 27% in performance, 33% in read energy and 33% in write energy, the drop of HM-W-Migr is lower with 12% in performance, 15% in read energy and 18% in write energy. Figure 8a shows the read/write request distribution on HBM and DRAM. We can find that the number of memory requests is increasing as the data size increases. Besides, compared to HM-W/O-Migr (i.e., W/O in Figure 8a), HM-W-Migr (i.e., W in Figure 8a) has a steady increase in the percentage of memory requests in HBM across all workloads.

#### 4.4.2. Sensitivity to Set-Get Operation Ratio

**Figure 9:**
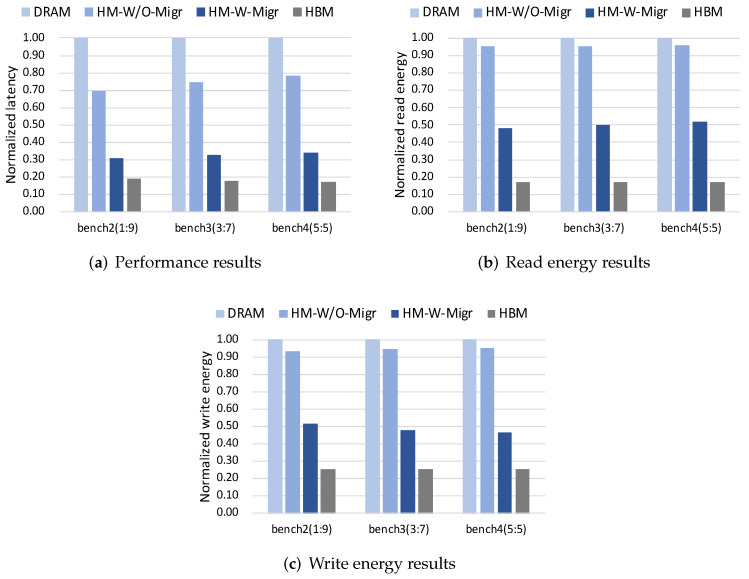
Normalized results across different set-get operation ratios.

Figure 9a–c show the performance, read energy and write energy results across three different operation ratios, respectively. When the percentage of set operation increases, HM-W/O-Migr shows a slight decrease in performance and energy improvement compared to the DRAM case. HM-W-Migr also shows the same results for performance and read energy, except for write energy. It can be seen from Figure 9c that, the write energy improvement of HM-W-Migr shows a slight increase when the portion of set operation becomes higher. Figure 8b describes the request distribution on HBM and DRAM across three operation ratios. It can be found that, the improvement in percentage of HBM write requests in HM-W-Migr gradually increases when the set operation ratio increases. In addition, the improvement in percentage of all HBM requests in HM-W-Migr are almost the same across three ratios. In summary, our proposed ADM method can improve both performance and energy results across different workloads.

## 5. Conclusions and Futuer Work

In this paper, we propose an application-oriented data migration method for in-memory database on the hybrid memory architecture. By establishing a hotness management scheme for die-stacked memory and DRAM, ADM designs the migrations into and out of HBM, and implements these two designs with new APIs by extending the existing in-memory database. We evaluate our ADM method using the Redis as a case study. Comprehensive experiments verify the effectiveness of ADM on performance improvement and energy consumption reduction. Specifically, compared to existing Redis database on hybrid memory architecture, our proposed ADM method can improve performance by 20%, read energy by 26% and write energy by 18% on average.

We expect to conduct two extensions based on our ADM method in the future work. On the one hand, we expect to improve the scalability of our approach. Our existing experiment takes Redis as case study for evaluation. Some other applications would be considered as cases to implement our ADM method. On the other hand, we would test some experimental data on real devices that would further verify the effectiveness of ADM.

## Figures and Tables

**Table 1:** Comparisons of various memories [16,17].

Feature	DDR4	DDR5	HBM2	HBM3
Max Pin BW	3.2 GB/s	6.4 GB/s	2.4 GB/s	6.4 GB/s
Max I/F BW	25.6 GB/s	51 GB/s	307 GB/s	819 GB/s
Pins/channel	380 pins	380 pins	2860 pins	2860 pins
Energy	5.1 pJ/bit	4 pJ/bit	0.7 pJ/bit	unpublished
Max capacity	128 GB	256 GB	8 GB	24 GB

**Table 2:** Memory configurations.

Caches	
L1 I-Cache	8-way, 3 cycles, 32 KB
L1 D-Cache	8-way, 4 cycles, 32 KB
L2 Cache	4-way, 7 cycles, 1 MB
L3 Cache	20-way, 27 cycles, 10 MB
**Conventional DRAM**	
Bus frequency	1.6 GHz
Channels	2
Banks	8
Bus width	64 bits/channel
tCAS-tRCD-tRP	22-22-22 bus cycles
**HBM Die-Stacked DRAM**	
Bus frequency	1 GHz
Channels	8
Banks	8
Bus width	128 bits/channel
tCAS-tRCD-tRP	7-7-7 bus cycles

**Table 3:** Workloads configurations.

Name	Data Size Ranges (Bytes)	Operation Ratios (Set:Get)
bench1	[1, 2000]	1:9
bench2	[1, 3000]	1:9
bench3	[1, 3000]	3:7
bench4	[1, 3000]	5:5
bench5	[1, 4000]	1:9

## References

[1] Redis. https://redis.io/.

[2] Memcached. http://memcached.org/.

[3] Ahn J., Yoo S., Mutlu O., Choi K. PIM-enabled instructions: A low-overhead, locality-aware processing-in-memory architecture. Proceedings of the 2015 ACM/IEEE 42nd Annual International Symposium on Computer Architecture (ISCA).

[4] Wulf W.A., McKee S.A. (1995). Hitting the memory wall: Implications of the obvious. ACM SIGARCH Comput. Archit. News.

[5] Jun H., Cho J., Lee K., Son H.Y., Kim K., Jin H., Kim K. Hbm (high bandwidth memory) dram technology and architecture. Proceedings of the 2017 IEEE International Memory Workshop (IMW).

[6] Pawlowski J.T. Hybrid memory cube (HMC). Proceedings of the 2011 IEEE Hot Chips 23 Symposium (HCS).

[7] Rogers B.M., Krishna A., Bell G.B., Vu K., Jiang X., Solihin Y. Scaling the bandwidth wall: Challenges in and avenues for CMP scaling. Proceedings of the 36th Annual International Symposium on Computer Architecture.

[8] Ramalingam S. HBM package integration: Technology trends, challenges and applications. Proceedings of the 2016 IEEE Hot Chips 28 Symposium (HCS).

[9] Vasilakis E., Papaefstathiou V., Trancoso P., Sourdis I. LLC-guided data migration in hybrid memory systems. Proceedings of the 2019 IEEE International Parallel and Distributed Processing Symposium (IPDPS).

[10] Sim J., Alameldeen A.R., Chishti Z., Wilkerson C., Kim H. Transparent hardware management of stacked dram as part of memory. Proceedings of the 2014 47th Annual IEEE/ACM International Symposium on Microarchitecture.

[11] Prodromou A., Meswani M., Jayasena N., Loh G., Tullsen D.M. Mempod: A clustered architecture for efficient and scalable migration in flat address space multi-level memories. Proceedings of the 2017 IEEE International Symposium on High Performance Computer Architecture (HPCA).

[12] In-Memory Database. https://en.wikipedia.org/wiki/In-memory_database.

[13] In-Memory Database. https://www.omnisci.com/technical-glossary/in-memory-database.

[14] Aerospick. https://aerospike.com/.

[15] Loh G.H. (2008). 3D-stacked memory architectures for multi-core processors. ACM SIGARCH Comput. Archit. News.

[16] SK Hynix Announces Development of HBM3 DRAM. https://news.skhynix.com/sk-hynix-announces-development-of-hbm3-dram/.

[17] Choosing between DDR4 and HBM in Memory-Intensive Applications. https://www.techdesignforums.com/practice/technique/choosing-between-ddr4-and-hbm-in-memory-intensive-applications/.

[18] Ryoo J.H., Meswani M.R., Prodromou A., John L.K. Silc-fm: Subblocked interleaved cache-like flat memory organization. Proceedings of the 2017 IEEE International Symposium on High Performance Computer Architecture (HPCA).

[19] Ramos S., Hoefler T. Capability models for manycore memory systems: A case-study with Xeon Phi KNL. Proceedings of the 2017 IEEE International Parallel and Distributed Processing Symposium (IPDPS).

[20] Kotra J.B., Zhang H., Alameldeen A.R., Wilkerson C., Kandemir M.T. Chameleon: A dynamically reconfigurable heterogeneous memory system. Proceedings of the 2018 51st Annual IEEE/ACM International Symposium on Microarchitecture (MICRO).

[21] Vasilakis E., Papaefstathiou V., Trancoso P., Sourdis I. Hybrid2: Combining caching and migration in hybrid memory systems. Proceedings of the 2020 IEEE International Symposium on High Performance Computer Architecture (HPCA).

[22] Memtier_Benchmark. https://github.com/RedisLabs/memtier_benchmark.

[23] Sanchez D., Kozyrakis C. (2013). ZSim: Fast and accurate microarchitectural simulation of thousand-core systems. ACM SIGARCH Comput. Archit. News.

[24] Li S., Yang Z., Reddy D., Srivastava A., Jacob B. (2020). DRAMsim3: A cycle-accurate, thermal-capable DRAM simulator. IEEE Comput. Archit. Lett..

[25] Cao W., Sahin S., Liu L., Bao X. Evaluation and analysis of in-memory key-value systems. Proceedings of the 2016 IEEE International Congress on Big Data (BigData Congress).

